# The Correlation Between Surgical Procedures and Quality of Life Among Sickle Cell Disease Patients: A Perspective Saudi Study

**DOI:** 10.7759/cureus.21367

**Published:** 2022-01-18

**Authors:** Ali J Al Saad, Rayan A Buhalim, Faisal A Al Jabr, Abdulaziz M Al Dehailan, Abdulaziz A Albahrani

**Affiliations:** 1 Clinical Neuroscience, King Faisal University, Al-Ahsa, SAU; 2 College of Medicine, King Faisal University, Al-Ahsa, SAU

**Keywords:** heath related quality of life, total joint arthroplasty, post-splenectomy, post cholecystectomy, surgeries, kingdom of saudi arabia (ksa), surgical operation, sickle cell anemia, psychological impacts, mental health and psychiatry

## Abstract

Introduction: Sickle cell disease (SCD) is among the common prevalent diseases in Saudi Arabia. It is associated with several complications that sometimes necessitate surgical procedures. Those patients can also have a lower quality of life (QoL) due to several reasons. Our aim in this study is to highlight the association between sociodemographic data, clinical data, and SCD-related surgeries in patients with their QoL.

Methods: A cross-sectional study was performed using a validated Arabic version of the World Health Organization QoL-BREF (WHOQOL-BREF) questionnaire distributed in electronic form. Male and female Arabic speakers (18+ years old) of Saudi origin were included in this study; those who did not meet these criteria were excluded.

Results: We distributed 309 questionnaires to the targeted subjects; however, only 204 met our inclusion criteria. Our findings revealed 135 female respondents and 69 male respondents. Interestingly, splenectomy was not significantly associated with a difference in all aspects of QoL in SCD patients. However, the data showed significantly lower respective mean scores for physical health (p=0.002 & p=0.022) and overall QoL (p=0.024 & p=0.042) for those who underwent cholecystectomy and hip arthroplasty. In contrast, shoulder arthroplasty appeared to be associated with significantly lower mean scores for physical health (T=-2.597; p=0.010) and the environmental aspect of QoL (T=-2.016; p=0.045).

Conclusion: Cholecystectomy, hip arthroplasty, and shoulder arthroplasty were significantly associated with lower QoL in SCD patients.

## Introduction

Sickle cell disease (SCD) is a major health problem in several countries [1]. This autosomal recessive disease results from a mutation in the β-globin chain due to the replacement of valine with glutamic acid at the sixth codon [2]. Worldwide, 300 million people carry the sickle cell trait (SCT), and 300,000 infants are born with SCD annually [3]. In the United States (US), SCD affects 72,000 people, and roughly 2 million are carriers [4]. In Saudi Arabia, the prevalence of SCD has not been widely studied. A community-based survey study found that the prevalence of SCD ranges from 6-145 per 10,000 people, with the highest prevalence in the Eastern Province [4-6]. This hereditary disease is pervasive in Saudi Arabia due to the high number of consanguineous marriages, which account for 56% of all marriages. Marriages between first cousins are the most common variation [4,5,7].

A vaso-occlusive crisis is the most common presentation of SCD in patients, and it is responsible for major organ damage [5]. One of the earliest and most commonly affected organs is the spleen [8]. Splenic damage increases susceptibility to encapsulated organisms (for example, *Streptococcus pneumoniae*, *Haemophilus influenzae*, and* Neisseria meningitidis*) and necessitates splenectomy and subsequent antibiotic prophylaxis [9,10]. Fortunately, splenectomy has been shown to improve the general and mental health of SCD patients [11]. Another commonly affected organ is the gallbladder, and SCD patients frequently experience cholelithiasis due to their excess bilirubin production [9]. Moreover, 20-50% of SCD patients suffer from femoral head osteonecrosis, altering their quality of life (QoL) and leading to debilitating consequences. Despite the high rate of perioperative complications, SCD patients with irreversible damage and compromised lifestyles are recommended to undergo early hip replacement surgery [12].

Measuring QoL in SCD patients could be critical to determining the efficacy of their treatments [13]. We hypothesized that performing SCD-related surgical procedures can improve patients’ mental well-being, social lives, and daily lives. Therefore, this study aimed to determine the correlations between QoL and SCD-related surgeries in Saudi patients.

## Materials and methods

Sample size and study procedure

Based on a study conducted during a Saudi premarital screening program, we could potentially access 1251 patients with SCD [14]. Consequently, we determined an ideal sample size of 294 patients, with a 5% margin of error and a 95% confidence interval(CI) [15]. We conducted a cross-sectional study using a convenient random sampling technique. An Arabic version of the abbreviated World Health Organization Quality of Life (WHOQOL-BREF) questionnaire was distributed to all SCD patients [16]. The data were collected from June to December 2020. The data included each patient’s age, gender, and other sociodemographic information as well as their clinical history of SCD.

Domain scores

We measured health-related QoL with the WHOQOL-BREF, which produced a QoL profile for each patient. We considered this profile to be valuable if the disease treatment option was palliative rather than curative. The questionnaire had 26 questions: two questions on general QoL and 24 questions from the original WHOQOL (WHOQOL-100) instrument. The WHOQOL-100 divides these 24 questions into four domains: physical health (seven items), psychological health (six items), social relationships (three items), and environment (eight items). The domain scores are distributed in a positive direction; the higher the domain scores, the better the QoL [17].

Statistical analysis

When appropriate, we presented the descriptive statistics using numbers, percentages, means, and standard deviations. Moreover, we conducted between-group comparisons, the chi-squared test, the independent t-test, and the one-way analysis of variance (ANOVA) test whenever it was appropriate to do so. We used p-values < 0.05 to determine statistical significance. We also performed a correlation procedure to assess the linear relationship between the overall QoL and its domains. We conducted all data analyses using the IBM SPSS Statistics for Windows, Version 21.0 (Released 2012l, IBM Corp., Armonk, New York).

Inclusion and exclusion criteria

We only included Saudi Arabic speakers over 18 years of age with SCD in this study. Hence, we excluded all other individuals.

Ethical considerations

The study was approved by the College of Medicine at King Faisal University, Hofuf, Saudi Arabia, and we ensured the participants’ confidentiality.

## Results

We distributed 309 questionnaires to the targeted subjects; 204 met our inclusion criteria. Table 1 presents the socio-demographic characteristics of the patients. The most common age group was less than 30 years (52.9%), with the majority being females (66.2%). Most of the respondents were living in the Eastern Province region (n=117, 57.4%). It was revealed that the prevalence of patients who were currently on medications was 72.1%, the proportion of the patients in support groups was 22.5%, while the percentage of patients admitted in the hospital in the last 12 months was 50.5%. However, the patients who take hydroxyurea, folic acid, narcotics, and analgesics were 39.7%, 30.4%, 3.4%, 5.9%, respectively. The prevalence of patients who underwent surgical procedures was 58.3%. Surgical procedures included cholecystectomy (32.8%), splenectomy (14.7%), hip arthroplasty (5.9%), and shoulder arthroplasty (1%).

**Table 1:** Sociodemographic characteristics of patients

Study data	Overall
	N (%)
		^(n = 204)^
	Age group		
	· < 30 years	108 (52.9%)
· ≥ 30 years	96 (47.1%)
Gender	
· Male	69 (33.8%)
· Female	135 (66.2%)
Residence region	
· Eastern region	117 (57.4%)
· Non-Eastern region	87 (42.6%)
Marital status	
· Never been married	121 (59.3%)
· Been married	78 (38.2%)
Support group	
· Yes	46 (22.5%)
· No	158 (77.5%)
Hospitalization during the previous 12 months	
· Yes	103 (50.5%)
· No	101 (49.5%)
Medications	
· Yes	147 (72.1%)
· No	57 (27.9%)
Hydroxyurea	
· Yes	81 (39.7%)
· No	123 (60.3%)
Folic acid	
· Yes	62 (30.4%)
· No	142 (69.6%)
Narcotics	
· Yes	7 (03.4%)
· No	197 (96.6%)
Analgesics	
· Yes	12 (05.9%)
· No	192 (94.1%)
Surgical procedure	
· Yes	119 (58.3%)
· No	85 (41.7%)
Splenectomy	
· Yes	30 (14.7%)
· No	174 (85.3%)
Cholecystectomy	
· Yes	67 (32.8%)
· No	137 (67.2%)
Hip arthroplasty	
· Yes	12 (05.9%)
· No	192 (94.1%)
Shoulder arthroplasty	
· Yes	2 (01.0%)
· No	202 (99.0%)

Table 2 shows the association between the sociodemographic characteristics and QoL. It was found that patients who were younger (<30 years) showed statistically significantly higher mean scores in domain 1 (T=2.379; p=0.018), domain 3 (T=3.392; p=0.001), domain 4 (T=3.198; p=0.002), and overall QoL (T=3.079; p=0.002). We also observed that females showed significantly higher mean scores in domain 3 (T=-2.318; p=0.021). Furthermore, those living in the Eastern region exhibited significantly higher mean scores in domain 1 (T=2.210; p=0.028), domain 3 (T=2.452; p=0.015), and overall QoL (T=2.029; p=0.044). Similarly, those who were living alone had significantly better mean scores in domain 3 (F=3.513; p=0.032), domain 4 (F=4.105; p=0.018), and overall QoL (F=3.235; p=0.041). In addition, those who had hospitalization during the last 12 months showed significantly lower mean scores in domain 1 (T=-6.535; p<0.001), domain 2 (T=-3.831; p<0.001), domain 3 (T=-2.979; p=0.003), domain 4 (T=-4.964; p<0.001), and overall QoL (T=-5.273; p<0.001).

**Table 2:** Statistical difference between sociodemographic characteristics versus overall QoL and its domain (n=204) Domain 1: Physical health; Domain 2: Psychological health; Domain 3: Social Relationship; Domain 4: Environmental; QoL: Quality of life. P-value has been calculated using Independent t-test **Significant at p<0.05 level.

Factor	Domain 1 Mean ± SD	Domain 2 Mean ± SD	Domain 3 Mean ± SD	Domain 4 Mean ± SD	Overall Mean ± SD
Age group					
<30 years	67.0 ± 15.6	68.5 ± 15.1	70.1 ± 17.1	66.3 ± 16.1	67.9 ± 13.5
≥30 years	61.8 ± 16.0	65.6 ± 15.8	61.0 ± 20.8	58.8 ± 17.2	61.8 ± 15.1
T-test	2.379	1.357	3.392	3.198	3.079
P-value	0.018 **	0.176	0.001 **	0.002 **	0.002 **
Gender					
Male	64.3 ± 17.8	65.7 ± 16.4	61.4 ± 20.7	60.1 ± 18.1	62.9 ± 15.8
Female	64.7 ± 15.1	67.9 ± 14.9	68.0 ± 18.4	64.1 ± 16.4	66.2 ± 13.8
T-test	-0.156	-0.963	-2.318	-1.579	-1.525
P-value	0.876	0.337	0.021 **	0.116	0.129
Residence region					
Eastern	66.7 ± 16.9	68.5 ± 16.5	68.7 ± 19.2	63.5 ± 17.1	66.8 ± 15.3
Non-Eastern	61.7 ± 12.3	65.2 ± 13.7	61.9 ± 19.2	61.8 ± 16.9	62.7 ± 13.2
T-test	2.210	1.519	2.452	0.708	2.029
P-value	0.028 **	0.130	0.015 **	0.480	0.044 **
Marital status					
Never been married	66.3 ± 14.7	67.4 ± 14.9	66.6 ± 17.4	64.2 ± 16.5	66.1 ± 13.2
Been married	62.0 ± 17.5	66.7 ± 16.3	64.7 ± 22.1	60.6 ± 17.7	63.5 ± 16.3
T-test	1.875	0.337	0.704	1.484	1.268
P-value	0.062	0.736	0.482	0.139	0.206
Hospitalization					
Yes	57.9 ± 13.3	63.1 ± 15.1	61.9 ± 18.6	57.2 ± 16.5	60.0 ± 13.4
No	71.3 ± 15.8	71.2 ± 14.8	69.8 ± 19.6	68.4 ± 15.8	70.2 ± 14.0
T-test	-6.535	-3.831	-2.979	-4.964	-5.273
P-value	<0.001 **	<0.001 **	0.003 **	<0.001 **	<0.001 **
Support group					
Yes	63.5 ± 17.5	66.9 ± 14.8	68.1 ± 18.9	65.0 ± 16.9	65.9 ± 14.3
No	64.9 ± 15.6	67.2 ± 15.6	65.1 ± 19.6	62.1 ± 17.1	64.8 ± 14.7
T-test	-0.516	-0.111	0.911	1.026	0.431
P-value	0.606	0.911	0.363	0.306	0.667

Table 3 highlights the differences in the mean scores of QoL and its domain in relation to the medications and surgical procedures done for the patients. It was found that those who were taking medications showed significantly lower mean scores in domain 1 (T=-4.334; p<0.001) and overall QoL (T=-2.685; p=0.002). We also observed that patients taking hydroxyurea exhibited a lower mean score in domain 1 (T=-2.915; p=0.004), while those who were taking Folic acid also exhibited a lower mean score in domain 1 (T=-2.841; p=0.005). Furthermore, those who were taking narcotics exhibited significantly less mean scores in domain 1 (T=-3.995; p<0.001), domain 2 (T=-2.431; p=0.016), domain 3 (T=-2.007; p=0.046), domain 4 (T=-2.792; p=0.006), and overall QoL (T=-3.229; p=0.001) while those who were taking analgesics showed significantly lower mean score in domain 1 (T=-3.041; p=0.003). Moreover, those who underwent surgical procedures showed significantly lower mean scores in domain 1 (T=-3.840; p<0.001), domain 4 (T=-2.211; p=0.028), and overall QoL (T=-2.427; p=0.016). Similarly, those who underwent cholecystectomy exhibited significantly lower mean scores in domain 1 (T=-3.062; p=0.002) and overall QoL (T=-2.273; p=0.024). Also, those with hip arthroplasty showed significantly less score in domain 1 (T=-2.315; p=0.022) and overall QoL (T=-2.043; p=0.042) while those with shoulder arthroplasty observed to have significantly less score in domain 1 (T=-2.597; p=0.010) and in domain 4 (T=-2.016; p=0.045). 

**Table 3:** Statistical differences between the QoL and its domain among the medications and surgical procedure done for the patients (n=204) Domain 1: Physical health; Domain 2: Psychological health; Domain 3: Social Relationship; Domain 4: Environmental; QoL: Quality of life. P-value has been calculated using independent t-test. ** Significant at p<0.05 level.

Factor	Domain 1 Mean ± SD	Domain 2 Mean ± SD	Domain 3 Mean ± SD	Domain 4 Mean ± SD	Overall Mean ± SD
Medications					
Yes	61.7 ± 15.5	65.8 ± 15.6	64.5 ± 19.0	61.5 ± 17.1	63.4 ± 14.6
No	72.0 ± 14.9	70.4 ± 14.7	69.1 ± 20.3	66.0 ± 16.5	69.4 ± 13.9
T-test	-4.334	-1.915	-1.517	-1.719	-2.685
P-value	<0.001 **	0.057	0.131	0.087	0.008 **
Hydroxyurea					
Yes	60.6 ± 13.2	65.6 ± 14.9	65.1 ± 18.6	60.2 ± 16.2	62.9 ± 13.2
No	67.2 ± 17.2	68.1 ± 15.7	66.3 ± 20.0	64.4 ± 17.4	66.5 ± 15.3
T-test	-2.915	-1.135	-0.425	-1.742	-1.741
P-value	0.004 **	0.258	0.671	0.083	0.083
Folic acid					
Yes	59.8 ± 15.9	64.7 ± 16.9	64.3 ± 20.8	61.8 ± 17.2	62.7 ± 15.7
No	66.6 ± 15.7	68.1 ± 14.7	66.5 ± 18.8	63.2 ± 17.0	66.1 ± 14.0
T-test	-2.841	-1.457	-0.735	-0.530	-1.555
P-value	0.005 **	0.147	0.463	0.597	0.122
Narcotics					
Yes	41.6 ± 9.01	53.3 ± 4.30	51.4 ± 23.6	45.4 ± 13.0	47.9 ± 9.83
No	65.4 ± 15.6	67.6 ± 15.5	66.3 ± 19.2	63.4 ± 16.9	65.7 ± 14.4
T-test	-3.995	-2.431	-2.007	-2.792	-3.229
P-value	<0.001 **	0.016 **	0.046 **	0.006 **	0.001 **
Analgesics					
Yes	51.2 ± 10.4	62.2 ± 13.5	61.7 ± 20.3	56.9 ± 9.54	57.9 ± 9.39
No	65.4 ± 15.9	67.4 ± 15.5	66.1 ± 19.4	63.1 ± 17.3	65.5 ± 14.8
T-test	-3.041	-1.131	-0.761	-1.229	-1.736
P-value	0.003 **	0.259	0.447	0.220	0.084
Surgical procedures					
Yes	61.0 ± 15.7	65.6 ± 14.7	64.8 ± 18.8	60.5 ± 17.1	62.9 ± 13.9
No	69.5 ± 15.2	69.2 ± 16.2	67.3 ± 20.4	65.8 ± 16.6	67.9 ± 15.1
T-test	-3.840	-1.655	-0.917	-2.211	-2.427
P-value	<0.001 **	0.099	0.360	0.028 **	0.016 **
Splenectomy					
Yes	63.1 ± 15.4	69.1 ± 14.4	67.8 ± 19.9	64.5 ± 17.9	66.1 ± 14.9
No	64.8 ± 16.1	66.8 ± 15.6	65.5 ± 19.4	62.4 ± 16.9	64.9 ± 14.6
T-test	-0.521	0.769	0.597	0.614	0.438
P-value	0.603	0.443	0.551	0.540	0.662
Cholecystectomy					
Yes	59.7 ± 15.4	64.3 ± 13.8	63.6 ± 18.0	59.4 ± 15.9	61.8 ± 12.6
No	66.9 ± 15.8	68.5 ± 16.0	66.9 ± 20.1	64.4 ± 17.4	66.7 ± 15.3
T-test	-3.062	-1.809	-1.149	-1.966	-2.273
P-value	0.002 **	0.072	0.252	0.051	0.024 **
Hip Arthroplasty					
Yes	54.3 ± 11.2	59.7 ± 17.4	55.6 ± 18.9	57.5 ± 18.2	56.8 ± 15.4
No	65.2 ± 16.1	67.6 ± 15.2	66.5 ± 19.3	63.1 ± 16.9	65.6 ± 14.4
T-test	-2.315	-1.717	-1.897	-1.097	-2.043
P-value	0.022 **	0.088	0.059	0.274	0.042 **
Shoulder arthroplasty					
Yes	35.7 ± 2.02	51.7 ± 2.36	56.7 ± 23.6	38.7 ± 5.30	45.7 ± 3.47
No	64.8 ± 15.8	67.3 ± 15.4	65.9 ± 19.4	62.9 ± 16.9	65.2 ± 14.5
T-test	-2.597	-1.425	-0.668	-2.016	-1.896
P-value	0.010 **	0.156	0.505	0.045 **	0.059

## Discussion

SCD is the most common monogenic disorder and a global health problem [18]. It occurs when sickle hemoglobin (HbS) replaces normal structural hemoglobin (HbA) [19]. Notably, the clinical sequels of SCD may negatively impact a patient’s QoL [20].

This study found that the younger patients had better QoL scores, which is supported by previous studies [20,21]. However, other studies have reported no statistical differences regarding psychological health and age in SCD patients [21]. This study determined that the female patients had higher social relationship scores, which contrasts with a previous study that could not identify any statistical differences between male and female patients [22]. Moreover, our findings support a previous study that reported lower QoL scores for patients who used narcotics [20]. This study associated hydroxyurea use with lower physical health scores, which contradicts a previous study [23] that found improvements in social life, pain recall, and general health. Similar to Ojelabi et al., we associated higher hospital admission with lower QoL scores in SCD patients [24]. One previous study revealed that comorbidities among SCD patients may significantly impact their QoL [20], and our study supports this conclusion.

SCD patients with recurrent acute splenic sequestration crises, hypersplenism, or splenic abscesses often undergo a splenectomy [9]. These indications can cause pain, which may lower QoL. If these patients undergo a splenectomy, this could lower the frequency of their hospitalization for blood transfusions, provide relief from an enlarged spleen and its symptoms, and prevent recurrent acute splenic sequestration crises and their complications [25]. However, we could not find an association between QoL and splenectomy. This might be because we did not measure QoL immediately after the operation. Also, recall bias may have influenced the results.

Our study contradicts Shurafa et al., who found that splenectomy enhanced the QoL of hematological disease patients in the domains of vitality, general health, and mental health [11]. However, they combined SCD and thalassemia, which may have caused their QoL findings to overlap. Our study showed that the patients who underwent a cholecystectomy had lower domain scores in physical health and overall QoL. In contrast, a previous study on the general population reported no significant differences in the patients’ QoL scores or their psychological symptoms [26]. This difference might be caused by the coexistence of SCD in our studied population, while the former study focused on the general population. Our study is the first to correlate cholecystectomy with QoL in SCD patients.

Another serious complication that can affect 20-50% of SCD patients is osteonecrosis of the femoral head, which can result in pain and restricted movement. Our findings significantly associated total hip arthroplasty with low QoL scores in SCD patients. However, one Saudi study suggested the opposite result [12]. Osteonecrosis can also affect the humeral head, which may necessitate shoulder arthroplasty. Our findings highlighted a significant relationship between low QoL scores and shoulder arthroplasty, while another study associated shoulder arthroplasty with high QoL scores [27].

Limitations

Aside from the limitations of the convenience sampling method, we acknowledge that it would be better to measure QoL preoperatively and postoperatively to minimize recall bias. Moreover, we did not combine the questionnaire with structured interviews, which could have enhanced data quality. Therefore, we recommend measuring QoL with structured interviews or group discussions to enhance the quality of the findings.

## Conclusions

This study revealed that cholecystectomy is the most common surgical procedure among SCD patients in Saudi Arabia. The findings associated surgical procedures with lower QoL scores and cholecystectomy with lower physical health and overall QoL scores in SCD patients. In addition, the findings significantly associated hip arthroplasty with lower physical health and overall QoL scores and shoulder arthroplasty with lower physical health and environmental QoL scores. However, the findings did not associate splenectomy with a statistical difference in QoL scores.
